# TOPIC CLUSTERS OF SUCCESSFUL AGING STUDIES: RESULTS OF A TOPIC MODELING APPROACH

**DOI:** 10.1093/geroni/igae098.3543

**Published:** 2024-12-31

**Authors:** Ha Neul Kim, Paul Freddolino

**Affiliations:** Michigan State University, East Lansing, Michigan, United States; Michigan State University, East Lansing, Michigan, United States

## Abstract

Literature regarding successful aging reflects a wide variety of fields and perspectives. Given the range of definitions and approaches found in the published literature, it is important to investigate clusters of topics studied over time. This study aimed to show the change of topic clusters within successful aging studies. The study used topic modeling methodology to analyze vast amounts of abstract data. Among publications collected from Scopus (4,458) and Web of Science (5,187), 5,610 publications were analyzed. Topic clusters were analyzed in two ways: by a) division of time (1960s – 1990s, 2000s, 2010s, 2020s) and b) all years combined. In the 1960s – 1990s, 11 topic clusters ranging from health to emotional well-being emerged without any dominant domain. In the 2000s, two clusters related to social support and health appeared as major clusters. In the 2010s, one topic cluster that included words related to health and social participation was the biggest. In the 2020s, emotional health and social participation appeared again as one of the major clusters and health-related topics started to diverge into subgroups like physical health and mental health. In all years of publications combined, the major cluster involved words that are related to either health or social domains. Results revealed that successful aging has been studied in many fields using multidimensional perspectives. The dominant categories were health and social domains. These findings suggest interprofessional practice, an interdisciplinary approach in research, and multi-sector involvement in policy.

